# Striatal dopaminergic alterations in individuals with copy number variants at the 22q11.2 genetic locus and their implications for psychosis risk: a [18F]-DOPA PET study

**DOI:** 10.1038/s41380-021-01108-y

**Published:** 2021-05-12

**Authors:** Maria Rogdaki, Céline Devroye, Mariasole Ciampoli, Mattia Veronese, Abhishekh H. Ashok, Robert A. McCutcheon, Sameer Jauhar, Ilaria Bonoldi, Maria Gudbrandsen, Eileen Daly, Therese van Amelsvoort, Marianne Van Den Bree, Michael J. Owen, Federico Turkheimer, Francesco Papaleo, Oliver D. Howes

**Affiliations:** 1 Department of Child and Adolescent Psychiatry, Institute of Psychiatry, Psychology and Neuroscience, King’s College, London, UK; 2 Department of Psychosis Studies, Institute of Psychiatry, Psychology and Neuroscience, King’s College, London, UK; 3 Psychiatric Imaging Group, MRC London Institute of Medical Sciences, Imperial College, London, UK; 4 Genetics of Cognition Laboratory, Neuroscience Area, Istituto Italiano di Tecnologia, Genova, Italy; 5 Centre for Neuroimaging Studies, Institute of Psychiatry, Psychology and Neuroscience, King’s College, London, UK; 6 Department of Radiology, University of Cambridge, Cambridge, UK; 7 Department of Radiology, Addenbrooke’s Hospital, Cambridge University Hospitals NHS Foundation Trust, Cambridge, UK; 8 Department of Psychological Medicine, Institute of Psychiatry, Psychology and Neuroscience, King’s College, London, UK; 9 South London and Maudsley NHS Foundation Trust, London, UK; 10 Department of Forensic and Neurodevelopmental Sciences, and the Sackler Institute for Translational Neurodevelopmental Sciences, Institute of Psychiatry, Psychology and Neuroscience, King’s College, London, UK; 11 Department of Psychiatry and Psychology, Maastricht University, Maastricht, The Netherlands; 12 Medical Research Council Centre for Neuropsychiatric Genetics and Genomics, Division of Psychological Medicine and Clinical Neurosciences, Cardiff University, Cardiff, UK

**Keywords:** Diseases, Schizophrenia

## Abstract

Dopaminergic dysregulation is one of the leading hypotheses for the pathoetiology underlying psychotic disorders such as schizophrenia. Molecular imaging studies have shown increased striatal dopamine synthesis capacity (DSC) in schizophrenia and people in the prodrome of psychosis. However, it is unclear if genetic risk for psychosis is associated with altered DSC. To investigate this, we recruited healthy controls and two antipsychotic naive groups of individuals with copy number variants, one with a genetic deletion at chromosome 22q11.2, and the other with a duplication at the same locus, who are at increased and decreased risk for psychosis, respectively. Fifty-nine individuals (21 with 22q11.2 deletion, 12 with the reciprocal duplication and 26 healthy controls) received clinical measures and [18F]-DOPA PET imaging to index striatal Ki^cer^. There was an inverse linear effect of copy number variant number on striatal Ki^cer^ value (*B* = −1.2 × 10^−3^, SE = 2 × 10^−4^, *p* < 0.001), with controls showing levels intermediate between the two variant groups. Striatal Ki^cer^ was significantly higher in the 22q11.2 deletion group compared to the healthy control (*p* < 0.001, Cohen’s *d* = 1.44) and 22q11.2 duplication (*p* < 0.001, Cohen’s *d* = 2) groups. Moreover, Ki^cer^ was positively correlated with the severity of psychosis-risk symptoms (*B* = 730.5, SE = 310.2, *p* < 0.05) and increased over time in the subject who went on to develop psychosis, but was not associated with anxiety or depressive symptoms. Our findings suggest that genetic risk for psychosis is associated with dopaminergic dysfunction and identify dopamine synthesis as a potential target for treatment or prevention of psychosis in 22q11.2 deletion carriers.

## Introduction

Schizophrenia is a highly heritable neurodevelopmental disorder [1–3] with a lifetime prevalence of 0.7% [4]. It is a leading cause of global disease burden in adults and is associated with high excess mortality rate [5]. Total costs for schizophrenia and related psychotic disorders are estimated at around €94 billion per annum across Europe [6]. Antipsychotic drugs, the main pharmacological treatment, show highly variable responses and are not effective in one-third of patients, leaving a substantial proportion of individuals with persistent impairments [7–9]. Improving the understanding of the neurobiology underlying schizophrenia is crucial to informing the development of new classes of treatment.

One of the main hypotheses for schizophrenia implicates dysregulation of the dopaminergic system [10, 11]. Over the last two decades, advances in molecular imaging have identified the nature of the dopamine (DA) dysfunction in patients with schizophrenia. Molecular imaging using radiolabelled DOPA, which is taken up into DA neurons and decarboxylated by aromatic amino acid decarboxylase (AADC) to give radiolabelled DA [12, 13], is used as an index of in vivo dopamine synthesis capacity (DSC) [12]. A meta-analysis of studies using this technique found increased striatal DSC in individuals with schizophrenia compared to controls with a large effect size [14].

Previous studies have shown elevated striatal DSC in people at clinical high risk for psychosis with a large effect size (Cohen’s *d* = 0.8) [15–18].Thus, elevated DSC at the striatal level is associated with both risk of psychosis and the development of symptoms. However, the degree to which dopaminergic alterations are due to trait risk for psychosis or reflect the symptomatic state is not clear from these studies [19]. Given the high heritability of schizophrenia [1, 2, 20], it has been hypothesized that one of the ways in which genetic risk for schizophrenia might be manifested is as increased DSC [21]. However, the results of studies examining DSC in first-degree relatives of people with schizophrenia are inconsistent, with one study showing increased DSC in unaffected first-degree relatives compared to controls [22] and a second one showing no difference in DCS between unaffected, predominantly dizygotic, twins for schizophrenia and controls [23]. This might reflect various methodological issues, including that unaffected first-degree relatives do not necessarily carry genetic risk variants for schizophrenia.

Given the importance of the question of whether genetic risk for schizophrenia is associated with DA dysfunction, we focused on individuals with a 1.5–3 megabase deletion at 22q11.2 locus. Carriers of this mutation are at greatly increased risk for developing psychosis [24, 25]; with a risk more than 40-fold greater than that in the general population [26], making this copy number variant one of the strongest genetic risk factors for developing schizophrenia and related psychotic disorders. In contrast, the reciprocal copy number variant, 22q11.2 duplication, has been associated with significantly reduced risk of schizophrenia compared to the general population, suggesting a possible protective role [27]. Subsequent studies have supported this initial finding of reduced risk of schizophrenia in 22q11.2 duplication [28–31], although some inconsistencies have been reported [32, 33]. Moreover, studies of peripheral DA metabolite levels suggest altered dopaminergic function in 22q11.2 deletion carriers [34, 35], consistent with the ubiquitous hemideletion in 22q11.2 deletion subjects of the Catechol-O-methyl-transferase, an enzyme involved in DA metabolism [36]. However, to our knowledge, no study has investigated DSC in 22q11.2 deletion carriers or any aspect of DA function in 22q11.2 duplication carriers.

In view of this, we tested the hypothesis that striatal DSC is related to genetic risk for psychosis and sub-clinical psychotic symptoms. We predicted that there would be a linear relationship between DSC and increasing genetic risk and with sub-clinical psychotic symptom severity, with 22q11.2 duplication carriers showing the lowest DSC and 22q11.2 deletion carriers showing the highest DSC.

## Methods and materials

Ethical permission was obtained by London-West London & GTAC Research Ethics Committee and the Administration of Radioactive Substances Advisory Committee. Following description of the study, informed written consent was given by all participants.

### Demographics

Twenty-one individuals with 22q11.2 deletion and 12 individuals with 22q11.2 duplication were recruited via support groups in Great Britain and Ireland, Clinical Genetics Clinics across the UK, and other cohorts in the UK and Netherlands. Inclusion criteria were: age above 18 years old, no history of schizophrenia or other psychotic disorder and no antipsychotic medication use. Confirmation of 22q11.2 deletion and duplication diagnosis was based on records from regional clinical genetics. Diagnosis for twenty of the individuals with 22q11.2 deletion was established by fluorescence in situ hybridization and for only one by comparative genomic hybridization (CGH) arrays. CGH arrays were used to establish the presence of the 22q11.2 duplication. Twenty-six healthy controls were recruited via local media for comparison. Inclusion criteria for controls were: no significant personal medical or psychiatric history, no family history of psychotic disorder, no concurrent use of psychotropic medication. Exclusion criteria for both groups: history of head trauma, significant medical disorder (unrelated to 22q11.2 deletion/duplication), significant illicit drug/alcohol use, any procedures, operations or medical conditions (e.g., pregnancy) that would compromise safety in scanning.

### Clinical assessments

The 22q11.2 deletion and duplication groups were assessed using the Comprehensive Assessment of At Risk Mental States (CAARMS), which measures sub-clinical psychotic-like symptoms, to determine if they met criteria for being at clinical high risk of psychosis [37]. All subjects received the Beck’s self-reported questionnaires for depression and anxiety [38]. Intellectual quotient was measured using an abbreviated version of the Wechsler Adult Intelligence scale (WAIS-III), consisting of four subtests [39].

### PET data acquisition

We used the approach previously described by Jauhar et al. [18]. All subjects were instructed to abstain from food and alcohol 12 h prior scanning and to refrain from tobacco smoking 4 h before scanning [40]. On the day of the scan, a urine drug screen verified no recent drug use and a negative pregnancy test was essential for all female participants. An hour before the scan, 150 mg carbidopa and 400 mg entacapone were administered orally to prevent the formation of radiolabelled metabolites [13]. All subjects underwent a 6-[18F] fluoro-L-DOPA ([18F]-DOPA) PET scan on the same Siemens Biograph 6 HiRez PET scanner (Siemens, Erlangen, Germany) in three-dimensional mode. Following a CT transmission scan for attenuation correction, ~150 MBq of [18]F-DOPA was administered by bolus intravenous injection within 30 s after the start of PET imaging.

### PET data analysis

Our main outcome was the influx rate constant (Ki^cer^) in the whole striatum. Given the evidence that dopaminergic alterations in schizophrenia may be more pronounced in associative striatum [14], we also conducted secondary analysis in functional subdivisions (associative, limbic, sensorimotor). To correct for head movement, nonattenuation-corrected dynamic images were denoised using a level 2, order 64 Battle–Lemarie wavelet filter [41], and individual frames were realigned to a single frame acquired 10 min after the [^18^F]-DOPA injection using a mutual information algorithm [42]. Transformation parameters were then applied to the corresponding attenuation-corrected frames, and the realigned frames were combined to create a movement-corrected dynamic image (from 6 to 95 min following [^18^F]-DOPA administration) for analysis.

Following motion correction, standardized regions of interest (ROIs) were defined bilaterally in the whole striatum and its functional subdivisions, and the cerebellar reference region in Montreal Neurologic Institute space [43]. Using statistical parametric mapping software (SPM8, http://fil.ion.ucl.ac.uk/spm) and in-house MATLAB-based scripts, we then co-registered an [18F]-DOPA template with the ROI map to each individual PET summation (add) image, allowing ROIs to be placed automatically on individual [^18^F]-DOPA PET images. This approach means that the volumes of the ROIs are transformed to fit the individual’s brain scan. A previous test rest study has shown good reliability with intra-class correlation coefficients > 0.84 for this approach [44]. In addition, since regional brain volumetric differences in 22q11.2 deletion and duplication have been described, for a subset of patients in which MRI imaging was available (*N* = 32), we also conducted MRI-based segmentation and compared this with the PET-based analysis (please see Supplementary material for more information). Structural MR imaging could not be obtained in all subjects because of contra-indications to MR imaging in a number of subjects, which are common in people with 22q11.2 deletions because of increased rates of surgery for cardiac conditions.

[^18^F]-DOPA uptake was calculated relative to the cerebellum, for each ROI using the Patlak graphic analysis modified for a reference tissue input function [45–47]. Further details of the image analysis are given in previous publications [18, 48, 49]. We also calculated the standardized uptake values in the cerebellum at 95 min as previously [18] to examine if there are any differences between groups.

### PET parametric mapping

We applied a previously established method [18, 49], in which the Ki^cer^ parametric images of the brain were constructed from movement-corrected images using a wavelet-based Patlak–Gjedde approach [47]. The parametric image for each subject was then normalized into Montreal Neurological Institute standard space, using the subjects’ PET summation image and the [18F]-DOPA template. Using SPM8 and a striatal mask [15], we performed statistical parametric mapping to compare striatal DSC between groups using an independent *t*-test. Results are presented after correction for multiple comparisons as applied in SPM8 (family-wise error (FWE) corrected).

### Statistical analysis

As our study was the first of its kind in this cohort, sample size was chosen based on previous [18F]-DOPA PET studies in individuals with psychosis [18, 48, 49]. Statistical analyses were performed using R version 3.3.2 and SPSS, version 23 (IBM SPSS Statistics for Macintosh, Version 24.0). Normality of distribution was assessed using Shapiro–Wilk test. Group comparisons of demographic variables were determined using univariate analysis of variance (ANOVA) or chi-square tests as appropriate. Our main analysis used a linear regression model with whole striatal Ki^cer^ as the dependent variable and group as a predictor to test our hypothesis that there would be a linear relationship between DSC and group in line with the genetic risk for psychosis, with 22q11.2 deletion carriers showing highest values and 22q11.2 duplication carriers showing lowest values. Where this was significant, we went on to conduct group comparisons using independent *t*-tests adjusted for multiple comparisons using Tukey’s tests. We repeated these analyses for the striatal functional subdivisions. We tested the relationship between Ki^cer^ and scores on clinical scales using a linear regression model with symptoms score as the dependent variable and Ki^cer^ as the predictor, and group as an additional factor. We also conducted exploratory sensitivity analyses, adjusting for injected activity, age, given their potential effect on Ki^cer^ [49], as well as for FSIQ, striatal volume and head motion and performed further sensitivity analyses excluding symptomatic 22q11.2 carriers and the 22q11.2 duplication carrier with co-morbid cerebral palsy in case this influenced findings. Subjects who scored two or less (meaning symptoms were absent or questionably present at most) on the CAARMS severity scale for positive symptoms were categorized as having no/minimal CAARMS symptoms, whilst the rest were categorized as being symptomatic and excluded for this sub-analysis.

## Results

### Demographics

No significant differences were observed between groups in gender and ethnicity, although the duplication group was older than the other groups (Table 1). As expected, individuals with 22q11.2 deletion had significantly lower FSIQ compared to the group of the reciprocal duplication and healthy controls (Table 1). All participants were antipsychotic naive. Two of the individuals with 22q11.2 were receiving selective serotonin reuptake inhibitors (citalopram 20 mg and fluoxetine 40 mg). One 22q11.2 duplication carrier had a history of cerebral palsy. None of the 22q11.2 deletion or duplication carriers met criteria to be at clinical high risk of psychosis [37]. Individuals with 22q11.2 deletion and duplication had higher self-reported scores in Beck’s depression and anxiety scales compared to healthy controls. Post hoc analysis showed that there were no statistically significant differences between the group of 22q11.2 deletion and the reciprocal duplication (*p* > 0.05).

**Table 1:** Demographic details.

Demographics				
Variable	22q11.2 deletion (*N* = 21)	22q11.2 duplication (*N* = 12)	Controls (*N* = 26)	*p* value
Gender (M: F)	7:14	7:5	11:15	0.38^a^
Age (years), mean (SD)	26.1 (7.73)	34.17(10.62)	26.12 (4.28)	0.004^b^
Ethnicity				0.78^a^
White	20	12	23	
Black	1	0	1	
Indian	0	0	1	
Other	0	0	1	
Tobacco				0.18^a^
Never	17	7	20	
Previously	0	3	2	
Currently	4	2	4	
CAARMS total, mean (SD)	23.1 (14.58)	16.08 (10.42)	N/A	0.154^b^
CAARMS positive, mean (SD)	4.62 (3.25)	3.25 (2.9)	N/A	0.24^b^
Beck’s depression scale score, mean (SD)	7.43 (7.92)	12 (9.79)	2.83 (5.37)	0.004^b^
Beck’s anxiety scale score, mean (SD)	18.52 (13.88)	21.17 (14.02)	7.17 (4.7)	0.001^b^
FSIQ, mean (SD)	80.43 (21.78)	98.5 (15.72)	130.63 (21.36)	<0.001^b^
Injected activity (MBq), mean (SD)	145.52 (2.18)	142.92 (8.5)	148.09 (7.1)	0.06^b^
Specific activity (MBq/μmol), mean (SD)	0.024 (0.008)	0.029 (0.008)	0.025 (0.015)	0.39^b^
Head motion (mm), mean (SD)	17.7 (9.44)	10.76 (5.45)	7.8 (3.19)	<0.001^b^

^a^*p* value was calculated using chi-square test.

^b^*p* value was calculated using ANOVA.

### Relationship between striatal dopamine synthesis capacity and genetic risk for psychosis

**Fig. 1:**
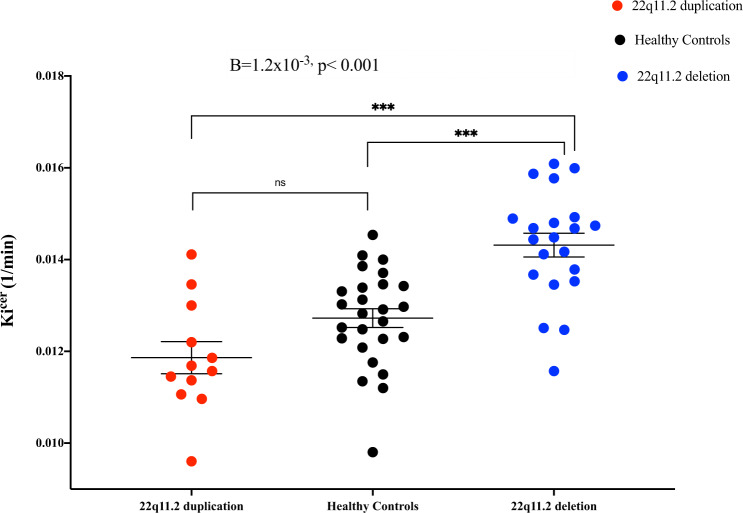
Dopamine synthesis capacity as measured by Ki^cer^ (1/min) in the whole striatum by group. Error bars indicate standard error of the mean. Dopamine synthesis and/or storage capacity was higher in individuals with 22q11.2 deletion compared to healthy controls and individuals with 22q11.2 duplication in the following order: 22q11.2 deletion > healthy controls > 22q11.2 duplication groups.

**Fig. 2:**
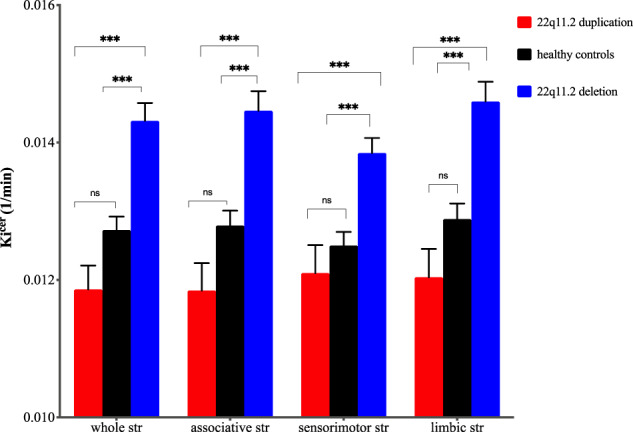
Mean dopamine synthesis capacity (Ki^cer^, 1/min) by group in whole striatum (str) and functional subdivisions. Error bars indicate standard error of the mean (***p < 0.001).

Using the linear regression model, we found that group significantly predicted DSC (as measured by Ki^cer^) levels in the whole striatum (*B* = −1.2 × 10^−3^, SE = 2 × 10^−4^, *p* < 0.001) (Fig. 1), and all striatal functional subdivisions (associative: *B* = −1.36 × 10^−3^, SE = 2.2 × 10^−4^, *p* < 0.001; sensorimotor: *B* = −9.4 × 10^−4^, SE = 2 × 10^−4^, *p* < 0.001; limbic: *B* = −1.3 × 10^−3^, SE = 2.3 × 10^−4^, *p* < 0.001) (Supplementary Table 1 and Fig. 2) with the following direction: 22q11.2 deletion > healthy controls > 22q11.2 duplication. Post hoc analyses, after adjusting for multiple comparisons, showed significantly greater whole striatal Ki^cer^ in the 22q11.2 deletion group relative to both healthy controls (*p* < 0.001, Cohen’s *d* = 1.44) and the 22q11.2 duplication group (*p* < 0.001, Cohen’s *d* = 2) (Supplementary Table 1). Similarly, post hoc analyses indicated greater Ki^cer^ in the 22q11.2 deletion group compared to the healthy control and 22q11.2 duplication groups in all striatal functional subdivisions (associative striatum: *p* < 0.001, Cohen’s *d* = 1.4; *p* < 0.001, Cohen’s *d* = 2, for healthy control and duplication comparisons, respectively; sensorimotor striatum: *p* < 0.001; Cohen’s *d* = 1.35, *p* < 0.001, Cohen’s *d* = 1.55, respectively; limbic striatum: *p* < 0.001 Cohen’s *d* = 1.7; *p* < 0.001, Cohen’s *d* = 2.2, respectively; Supplementary Table 1 and Fig. 2).

As, by chance, the duplication group was older than the other groups, we conducted exploratory analyses to determine if age influenced our findings. Age showed no relationship with Ki^cer^ (*B* = −1.21 × 10^−5^, SE = 2.05 × 10^5^, *p* = 0.6). Moreover, when age was added to the model, group remained a significant predictor of Ki^cer^ for the whole striatum (*B* = −1.23 × 10^−3^, SE = 2.16 × 10^−4^, *p* < 0.001) and its subdivisions (associative; *B* = −1.3 × 10^−3^, SE = 2.4 × 10^−4^, *p* < 0.001; sensorimotor *B* = −8.65 × 10^−4^, SE = 2.2 × 10^−4^, *p* < 0.001; limbic: *B* = −1.35 × 10^−3^, SE = 2.5 × 10^−4^, *p* < 0.001).

Further analysis was also performed to determine if the dose of injected activity influenced our findings. There was no association between injected activity and Ki^cer^ (*B* = 6.14 × 10^−6^, SE = 2.35 × 10^−5^, *p* = 0.8). In addition, when we added injected activity to the model, group continued to significantly predict Ki^cer^ levels in the whole striatum (*B* = −1.27 × 10^−3^, SE = 2.05 × 10^−4^, *p* < 0.001) and its subdivisions (associative; *B* = −1.35 × 10^−3^, SE = 2.25 × 10^−4^, *p* < 0.001; sensorimotor; *B* = −9.5 × 10^−4^, SE = 2.07 × 10^−4^, *p* < 0.001; limbic; *B* = −1.34 × 10^−3^, SE = 2.3 × 10^−4^, *p* < 0.001).

As previously noted, FSIQ was, unsurprisingly, lower in individuals with 22q11.2 deletion compared to the groups of healthy controls and 22q11.2 duplication. Therefore, we tested whether FSIQ had an effect on our results. We found no relationship between FSIQ and Ki^cer^ (*B* = −7.33 × 10^−6^, SE = 5.38 × 10^−6^, *p* = 0.18). In addition, when we added FSIQ to the model, group remained a significant predictor of Ki^cer^ in the whole striatum (*B* = −1.17 × 10^−3^, SE = 2.18 × 10^−4^, *p* < 0.001) and its subdivisions (associative; *B* = −1.21 × 10^−3^, SE = 2.43 × 10^−4^, *p* < 0.001; sensorimotor; *B* = −8.3 × 10^−4^, SE = 2.24 × 10^−4^, *p* < 0.001; limbic; *B* = −1.19 × 10^−3^, SE = 2.5 × 10^−4^, *p* < 0.001).

In addition, as head motion was significantly greater in the individuals with 22q11.2 deletion and duplications compared to healthy controls, we examined whether the above factor influenced our results. There was no association between head motion and Ki^cer^ (*B* = 2.19 × 10^−5^, SE = 2.09 × 10^−5^, *p* = 0.3). Furthermore, the addition of head motion to the model did not alter our results and group predicted significantly Ki^cer^ in the whole striatum (*B* = −1.18 × 10^−3^, SE = 2.2 × 10^−4^, *p* < 0.001) and its subdivisions (associative; *B* = −1.26 × 10^−3^, SE = 2.4 × 10^−4^, *p* < 0.001; sensorimotor; *B* = −8.96 × 10^−4^, SE = 2.23 × 10^−4^, *p* < 0.001; limbic; *B* = −1.21 × 10^−3^, SE = 2.48 × 10^−4^, *p* < 0.001).

To exclude a potential effect of group on blood flow and peripheral metabolism of DOPA, we examined the reference region (cerebellum) to test whether there was any difference in the standardized uptake value at 95 min between the three groups, as previously described [50], and found no significant differences (F(2, 56) = 2.2, *p* = 0.12) (Supplementary Fig. 1).

Furthermore, when we compared PET- and MRI-based segmentation for the subgroup of individuals of our cohort that MRI was available, analysis revealed similar results (please see Supplementary results, Supplementary Figs. 2 and 3 and Supplementary Table 2).

Moreover, all results remained significant in a further sensitivity analysis excluding the individual with 22q11.2 duplication with cerebral palsy (Supplementary Table 3). To determine if our findings were driven by the inclusion of symptomatic volunteers, we conducted a sensitivity analysis restricted to volunteers in the 22q11.2 group with no/minimal psychotic symptoms (*n* = 12). The effect of group on whole striatal Ki^cer^ value remained significant relative to the healthy control and the 22q11.2 duplication groups in this analysis (Supplementary Table 4).

One individual of the 22q11.2 deletion group developed schizophrenia 6 months following the initial PET scan. We were able to acquire PET data at the second-time point (2 months after his diagnosis), when he was treated with a low dose of risperidone (1 mg/day). At follow-up, Ki^cer^ showed a 6.2% increase from baseline (at baseline: Ki^cer^ = 0.016 min^−1^, and follow-up: Ki^cer^ = 0.017 min^−1^) (Supplementary Fig. 4).

### Voxel-based analyses

**Fig. 3:**
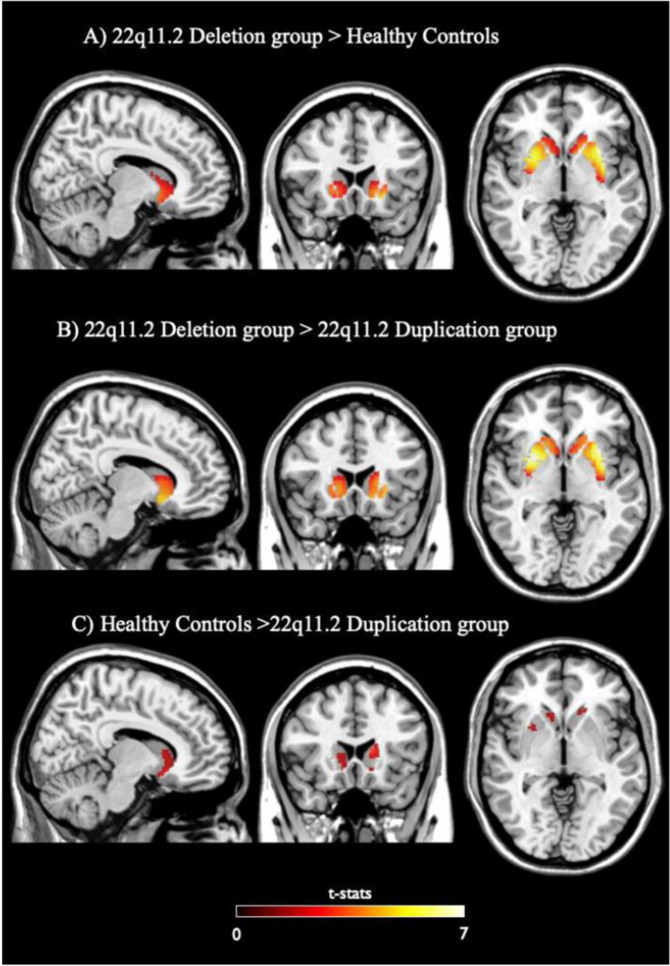
Voxelwise comparison of Striatal Dopamine Synthesis Capacity between groups. **A** Regions of significantly higher striatal Ki^cer^, relative to healthy controls (*N* = 26), in individuals with 22q11.2 deletion (*N* = 21). The most significant statistical increases had peaks in the left (peak MNI coordinates *x* = −24, *y* = 12, *z* = 4; *p*_uncorr_ = 0.001) and right putamen (peak MNI coordinates *x* = 28, *y* = 10, *z* = 4; *p*_uncorr_ = 0.016). **B** Regions of significantly higher striatal Ki^cer^, relative to 22q11.2 duplication group (*N* = 11), in 22q11.2 deletion subjects (*N* = 21). The most significant increases had peaks in the right (peak MNI coordinates *x* = 28, *y* = 8, *z* = −6; *p*_uncorr_ = 0.002) and left putamen (peak MNI coordinates *x* = −28, *y* = 12, *z* = −6; *p*_uncorr_ = 0.003). **C** Regions of significantly lower striatal Ki^cer^, relative to healthy controls (*N* = 26), in individuals with 22q11.2 duplication (*N* = 11). The most significant reductions had peaks in the left (peak MNI coordinates *x* = −12, *y* = 16, *z* = 12; *p* = 0.001) and right caudate nuclei (peak MNI coordinates *x* = 10, *y* = 14, *z* = 12; *p*_uncorr_ = 0.008). None of these voxel peaks survived correction for multiple comparisons using FWE. All maps refer to *t*-stats statistics threshold at *p* < 0.05 uncorrected.

We found significantly greater Ki^cer^ in the 22q11.2 deletion group relative to the controls in two voxel clusters with their foci in left and right putamen, both within the associative subdivision of the striatum (Fig. 3A; *p* < 0.05 corrected for multiple comparisons using the FWE rate method). There was also a significantly greater Ki^cer^ in the 22q11.2 deletion group relative to the duplication group in two voxel clusters within the right and left putamen (Fig. 3B; *p* < 0.05 FWE corrected for multiple comparisons). Furthermore, the voxel-based analysis identified a greater Ki^cer^ in the healthy control group relative to the 22q11.2 duplication group in two voxel clusters within the left and right caudate (Fig. 3C; *p* < 0.05 uncorrected), but this did not survive correction for multiple comparisons. The control group > 22q11.2 deletion group, the 22q11.2 duplication group > 22q11.2 deletion group and 22q11.2 duplication group > control group contrasts revealed no significant differences, even at an uncorrected statistical threshold (*p* < 0.05).

In summary, the region-of-interest and voxel-based analyses show that striatal DSC is higher in individuals with the 22q11.2 deletion compared to healthy controls and individuals with the 22q11.2 duplication.

### Relationship between dopaminergic function and sub-clinical positive symptoms in individuals with 22q11.2 deletion/duplication

**Fig. 4:**
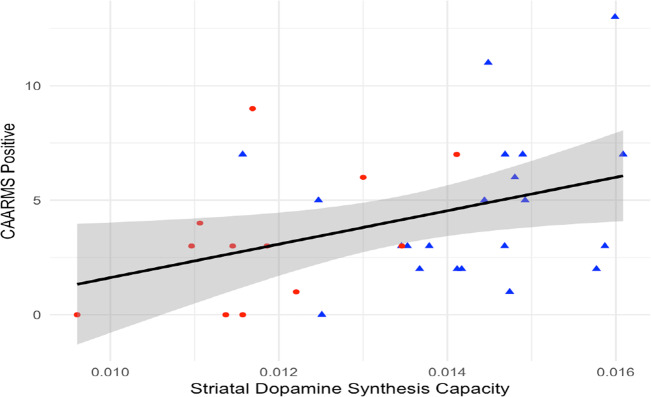
Relationship between CAARMS positive score and DSC in striatum (Ki^cer^, 1/min). Blue triangles and red circles indicate individuals with 22q11.2 deletion and 22q11.2 duplication, respectively. There was a positive relationship between CAARMS positive score and striatal Ki^cer^ (*R*^2^ = 0.15, *p* < 0.05).

We then tested the hypothesis that 22q11.2-dependent modulation of striatal dopaminergic tone might correlate with clinical phenotypes. There was a significant direct relationship between whole striatal Ki^cer^ and mean positive symptom severity ratings, as measured with the CAARMS [37] in the groups with copy number variants on 22q11.2 locus (*B* = 730.5, SE = 310.2, *p* = 0.025) (Fig. 4). When group status (duplication or deletion) was added to the model, Ki^cer^ remained significant (*p* = 0.03), but group was not predictive of CAARMS mean positive symptom domain score (*p* = 0.58), and there was no significant group × Ki^cer^ interaction (*p* = 0.65).

Anxiety and depression symptoms are also reported in the prodrome to psychosis in individuals with 22q11.2 deletion [51–53]. Thus, to test the specificity of our findings to positive symptoms, we also examined the association between Ki^cer^ levels and Beck’s Anxiety and Depression Scales. No significant relationships were found with mean scores on either the anxiety or depression scales (*B* = 179.33, SE = 1472.37, *p* = 0.9; *B* = −1364.03, SE = 906.67, *p* = 0.14, respectively).

Overall, these data suggest that there is a direct correlation of striatal DSC with sub-clinical psychotic symptoms, but not mood or anxiety symptoms.

## Discussion

Our study showed that striatal Ki^cer^ is higher in individuals with 22q11.2 deletion compared to healthy controls, who, in turn, have higher striatal Ki^cer^ than individuals with the reciprocal duplication. Moreover, Ki^cer^ is directly correlated with sub-clinical positive psychotic symptom severity.

The 22q11.2 deletion is the single largest genetic risk factor for schizophrenia and other psychotic disorders [26], whilst the 22q11.2 duplication is associated with reduced risk [27, 30, 31, 54]. Our findings are thus relevant to understanding the mechanism underlying psychosis. They extend previous findings of elevated DA synthesis and release capacity in people at clinical high risk for psychosis [15, 16, 55], by showing that DA alterations predate the clinical high-risk phase in people with a confirmed genetic high risk for schizophrenia. The 22q11.2 deletion carriers were young, and still in the peak age risk group for the onset of schizophrenia but had not yet developed a psychotic disorder or met clinical high-risk criteria. Thus, they remain at high risk of developing a psychotic disorder, and, based on previous studies [56], we anticipate that about 40% will go on to develop a schizophreniform disorder. Furthermore, our findings suggest lower Ki^cer^ in the 22q11.2 duplication carriers could be a mechanism underlying the lower risk for schizophrenia seen in this group.

In our study, the magnitude of the increase in DSC was comparable in all functional striatal subdivisions, which is contrary to the results of the most recent meta-analysis in schizophrenia showing greater dopaminergic alterations in the dorsal striatum than in the limbic striatum [14]. Taken with our findings across the striatum, this may point to differences in pathophysiology between idiopathic schizophrenia and that associated with 22q11.2 deletion. Another potential explanation may be that, whilst the 22q11.2 deletion has an effect in all functional subdivisions, subsequent stressors lead to alterations becoming more marked in the dorsal striatum in the 22q11.2 carriers who go on to develop schizophrenia, in line with stress-diathesis models of psychosis [57]. Longitudinal studies in genetic risk groups for developing psychosis are warranted to test this hypothesis.

Strengths of the study include that the participants were antipsychotic naive and the 22q11.2 deletion carriers were within the age range of risk for developing psychosis, factors that are both significant in the interpretation of our results. Our study was cross-sectional, therefore does not prove causality. Thus, follow-up of our sample is required to determine how Ki^cer^ links to the subsequent development of psychosis. Moreover, individuals with 22q11.2 duplication were significantly older compared to the group of 22q11.2 deletion and healthy controls, but, we, nevertheless, found no association between age and Ki^cer^, and when age was added to our model, results remained unchanged. In addition, PET regional volume analysis showed that striatal volume differed between groups, with individuals with 22q11.2 deletion having smaller striatal volume compared to other groups. This could lead to underestimation of Ki^cer^ estimates due to partial volume effects, therefore cannot account for the elevation we observed. Moreover, there was no relationship between striatal volume and Ki^cer^ estimates. Similarly, we noted greater head movement in the 22q11.2 deletion and duplication groups. We applied a motion correction algorithm to adjust for between frame motions. Notwithstanding this, greater motion is unlikely to explain the elevated Ki^cer^ in the deletion group as it would also lead to underestimation of Ki^cer^. Furthermore, we showed no association between head motion and Ki^cer^.

To our knowledge this is the first in vivo imaging study to examine striatal DA function using [^18^F]-DOPA in individuals with either a 22q11.2 deletion or 22q11.2 duplication. Previously, Butcher et al. [58] conducted a PET study using ^11^C-dihydrotetrabenazine (^11^C-DTBZ) in 13 individuals with 22q11.2 deletion, seven of whom were treated with antipsychotic medication. This radiotracer binds to the vesicular monoamine transporter (VMAT2), which transports DA into storage vesicles ready for release [59, 60]. Results showed elevated ^11^C-DTBZ binding in 22q11.2 carriers, indicating elevated VMAT2 levels and/or synaptic vesicle density. In contrast, DA receptor availability is not altered in 22q11.2 carriers relative to healthy controls [61].

Boot et al. [34] conducted a challenge study in individuals with 22q11 deletion with alpha-methyl paratyrosine (AMPT), a reversible inhibitor of the hydroxylation of tyrosine, the first step for catecholamine biosynthesis. At baseline, they found that levels of urinary DA were increased and homovanillic acid (HVA), the primary DA metabolite, was lower in the group of 22q11 deletion compared to controls. After AMPT, urinary and plasma HVA levels were found to be statistically lower in the 22q11 deletion group relative to the control group. Moreover, the ratio of DA/HVA was increased in subjects with 22q11 deletion both prior to AMPT and following AMPT points. Taken together, these prior findings indicate higher DA levels and reduced metabolism of DA in 22q11.2 carriers.

[18F]-DOPA PET indexes the uptake and conversion of [18F]-DOPA to [18F]-dopamine by aromatic aminoacid decarboxylase (AADC) in DA neurons, and the storage of [18F]-dopamine in synaptic vesicles [12]. Thus, the higher Ki^cer^ we report in the 22q11.2 deletion carriers could represent an increase in any one of these processes. However, when taken with prior findings that DA metabolite levels are reduced, and that VMAT2 levels are increased [58], a plausible interpretation of our findings in 22q11.2 deletion carriers is that there is increased synthesis and storage of [18F]-dopamine in vesicles due to reduced catabolism and increased VMAT2 levels in the terminal. This suggests that targeting DA synthesis or storage could be a novel treatment strategy for psychosis in 22q11.2 carriers [62]. Preclinical studies using animal models of 22q11.2 deletion and reciprocal duplication are warranted to further define the neurochemical substrates of dopaminergic alterations resulting from mutations in the 22q11.2 locus.

Our findings link increased striatal DA synthesis and storage capacity to a known genetic risk variant that moderates risk for psychotic disorders. Taken with other evidence of links between [18F]-DOPA and risk of schizophrenia [15, 63], our results suggest that [18F]-DOPA imaging could have potential as a biomarker to identify people at elevated risk of schizophrenia, although follow-up of our cohort is required to confirm this.

Whilst our results support the hypothesis of a trait component to DA alterations in schizophrenia, this does not rule out a state component as well. Supporting this, it should be noted that about 50% of the variance in striatal Ki^cer^ is attributable to unique environmental factors [64], patients in an acute relapse show greater striatal DA release than remitted patients [65], and we found an association between greater Ki^cer^ and greater symptom severity. The fact that we found no association between Ki^cer^ and depressive or anxiety symptoms indicates that this is specific to sub-clinical psychosis-risk symptoms and not an association with general morbidity. Moreover, in our cohort, the 22q11.2 deletion carrier who was re-scanned after developing psychosis showed increased Ki^cer^ compared to his baseline value, which is in agreement with studies of people at clinical high risk who transition to psychosis and comparisons between patients with schizophrenia in an acute relapse relative to remitted patients [65, 66]. However, further follow-up is required to determine if increases are seen in other subjects who develop psychosis and to exclude an effect of antipsychotic treatment [48, 67].

Lastly, individuals with 22q11.2 duplication had higher Beck’s depression and anxiety scale scores compared to the healthy control group, although there were no statistically significant differences in these measures between the two groups with copy number variants at the 22q11.2 locus. Anxiety and depressive symptoms are also commonly reported in people at clinical high risk for psychosis, although no relationship with striatal DSC has been reported [16], which is consistent with our results. Our findings of a high prevalence of anxiety and depressive symptoms in the 22q11.2 duplication carriers adds to evidence, albeit limited, for an increased prevalence of anxiety and mood disorders in people with 22q11.2 duplication [28]. Given the relatively small number of study participants in our group, our results highlight the need for further studies on the prevalence of neuropsychiatric symptoms in carriers of this mutation.

## Conclusions

We demonstrated that striatal DSC is higher in antipsychotic naive individuals with 22q11.2 deletion relative to controls, who, in turn, have higher striatal Ki^cer^ than individuals with the reciprocal duplication and is directly associated with sub-clinical psychosis risk but not anxiety or depressive symptom severity. This suggests that altered striatal DSC may be a mechanism mediating genetic risk for psychosis and the development of sub-clinical psychotic symptoms, suggesting potential targets for the development of new treatments and predictive biomarkers that can be applied to identify and prevent psychosis.

### Supplementary information

Supplementary Material
