# Supplementary material for: Striatal dopaminergic alterations in individuals with copy number variants at the 22q11.2 genetic locus and their implications for psychosis risk: a [18F]-DOPA PET study
Source: Mol Psychiatry. 2021 May 12;28(5):1995–2006. doi: 10.1038/s41380-021-01108-y (PMC10575769; doi:10.1038/s41380-021-01108-y)
Supplement: Supplementary file 1 — Supplementary Material [file 41380_2021_1108_MOESM1_ESM.docx]

**Supplementary Material**

**Striatal dopaminergic alterations in individuals with copy number variants at the 22q11.2 genetic locus and their implications for psychosis risk: a [18F**]**-DOPA** **PET study**

Maria Rogdaki, Céline Devroye, Mariasole Ciampoli, Mattia Veronese, Abhishekh Ashok, Robert A McCutcheon, Sameer Jauhar, Ilaria Bonoldi, Maria Gudbrandsen, Eileen Daily, Therese van Amelsvoort, Marianne Van Den Bree, Michael J Owen, Federico Turkheimer, Francesco Papaleo, Oliver D Howes

**Corresponding author:** maria.rogdaki@kcl.ac.uk

This file includes:

**Supplementary Methods:** Comparison of MRI to PET based segmentation for a subgroup of the cohort.

**Supplementary Results:** Results from the comparison of MRI to PET based segmentation for a subgroup of the cohort.

**Supplementary Figures**

**Supplementary Figure 1.** [18F]-DOPA Standardised Uptake Value (SUV) in the cerebellum.

**Supplementary Figure 2**. Comparison of striatal segmentation, using PET-based atlas co-registration and MRI-based atlas co-registration.

**Supplementary Figure 3.** Comparison of Ki^cer^ estimates using PET-based and MRI-based atlas co-registration in 22q11.2 deletion carriers and healthy controls.

**Supplementary Figure 4.** Individual Ki^cer^ estimates (1/min) at baseline and follow up for the individual with 22q11.2 deletion who developed schizophrenia.

**Supplementary Tables**

**Supplementary Table 1** Mean (SD) Ki^cer^ estimates for the whole striatum and for the striatal subdivisions by group.

**Supplementary Table 2.** Striatal volume as quantified using regional PET volume analysis in the groups of 22q11.2 deletion, 22q11.2 duplication and healthy controls.

**Supplementary Table 3.** Sensitivity analysis: Mean (SD) Ki^cer^ estimates for the whole striatum and for the striatal Subdivisions by study group after the exclusion of the individual with 22q11.2 duplication and co-morbid cerebral palsy.

**Supplementary Table 4.** Mean (SD) Ki^cer^ estimates for the whole striatum and for the striatal subdivisions for the group of 22q1.2 deletion with no/minimal CAARMS positive symptoms, healthy controls and the individuals with 22q11.2 duplication.

***Supplementary methods***

**Comparison of MRI to PET based segmentation for a subgroup of the cohort.**

Segmentation of regions of interest for both striatum and cerebellum was based on brain atlases. The brain atlases were spatially transformed from their MNI space into the individual’s PET space to obtain subject specific segmentation (PET-based segmentation). This is a commonly used approach for PET image analysis even in presence of neurodegenerative conditions^1^ and has been consistently applied for [18F]-DOPA PET quantification in psychiatric^2, 3^ and other neurological conditions ^4^.

For the subset of patients in which MRI imaging was available (N=32), the brain atlases were nonlinearly warped from their MNI space into the individual’s MRI space to obtain subject specific segmentation (MRI-based segmentation). These atlases were then linearly co-registered from their individual’s MRI space into the individual motion-corrected PET space consistently with the PET-based segmentation.

**Supplementary Results**

When comparing striatal segmentation, using PET-based atlas co-registration and the MRI-based atlas co-registration, we found that both approaches offer very similar delineation of the striatum, providing further support for the use of the PET based co-registration in our study (Supplementary Figure 3).

MRI-based segmentation led to mean (±SD) differences in Ki^cer^ estimates of around 4%±7% when individual MRIs were used to normalise the atlases to the individual FDOPA summed images, as compared to PET-based segmentation estimates. Nevertheless, Ki^cer^ estimates with the two segmentation methods remained highly correlated (Pearson’s r = 0.80, p<0.001) (Supplementary Figure 4A). Notably, results with MRI based segmentation showed a similar higher Ki^cer^ in the 22q11.2 relative to control group (whole striatal Ki^cer^ in 22q11.2 deletion versus healthy controls: PET-based +11% higher than controls, p<0.01; MRI-based +9% higher than controls, p=0.02) (Supplementary Figure 4B).

An analysis of the ROI volumes used in the PET-based segmentation showed that striatal volume was significantly different between groups (*F*(2,55)=4.7, p=0.013*).* Post hoc analysis revealed significantly decreased striatal volume in the group of 22q11.2 deletion compared to the groups of healthy control (p=0.021), but not between 22q11.2 deletion and 22q11.2 duplication (p=0.9) or between 22q11.2 duplication and healthy controls (p=0.07) (Supplementary Table 4). We also explored whether striatal volume had an influence on our results: when we added striatal volume to the statistical model, group continued to significantly predict Ki^cer^ levels in the whole striatum (B=-1.24x10^-3^, SE=2.1x10^-4^, p<0.001) and its subdivisions (associative; B=-1.36x10^-3^, SE=2.25x10^-4^, p<0.001; sensorimotor; B=-9.4x10^-4^, SE=2.06x10^-4^, p<0.001; limbic ; B=-1.34x10^-3^, SE=2.32x10^-4^, p<0.001).

**Supplementary Figures:**

**Supplementary Figure 1.** [18F]-DOPA Standardised Uptake Value (SUV) in the cerebellum. There was no difference in [18F]-DOPA SUV in the cerebellum (p=0.12) between groups. Error bars indicate standard error.

**Supplementary Figure 2**. Representative comparison of striatal segmentation, using PET-based atlas co-registration (red) and the MRI-based atlas co-registration (blue). In both PET and MRI co-registration, a striatal mask was used based on the anatomical delineation of the striatum described by Martinez and colleagues^5^*.*Image on the background (grey) refers to individual structural MRI and [18F]-DOPA PET merged scans. PET-based segmentation provides a striatal volume that is 26%±8% bigger than the MRI-based one, mainly due to PET tracer spill over into surrounding tissues. However, the majority of the voxels identified by the two segmentation methods overlaps, showing that the two approaches provide very similar delineation of the striatum and supporting our use of the PET based co-registration.


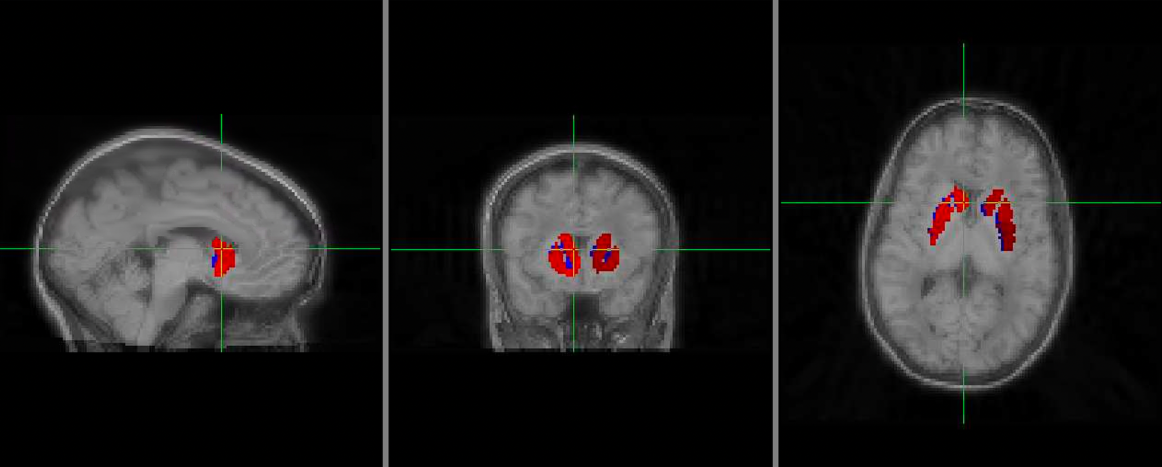


**Supplementary Figure 3.** Comparison of whole striatal Ki^cer^ estimates using PET-based and MRI-based atlas co-registrations in 22q11.2 deletion carriers (orange) and healthy controls (blue).

**A)** There is close agreement between the two methods (r=0.8, p<0.001), although the MRI based method returns slightly higher values for most subjects.

**B)** Whole striatal mean Ki^cer^ is significantly greater in the 22q11.2 group relative to the control group when the PET based (p< 0.01) or MRI based (p=0.02) co-registration is used.

**Supplementary Figure 4.** Individual Ki^cer^ values (1/min) at baseline and follow up for the individual with 22q11.2 deletion who developed schizophrenia.


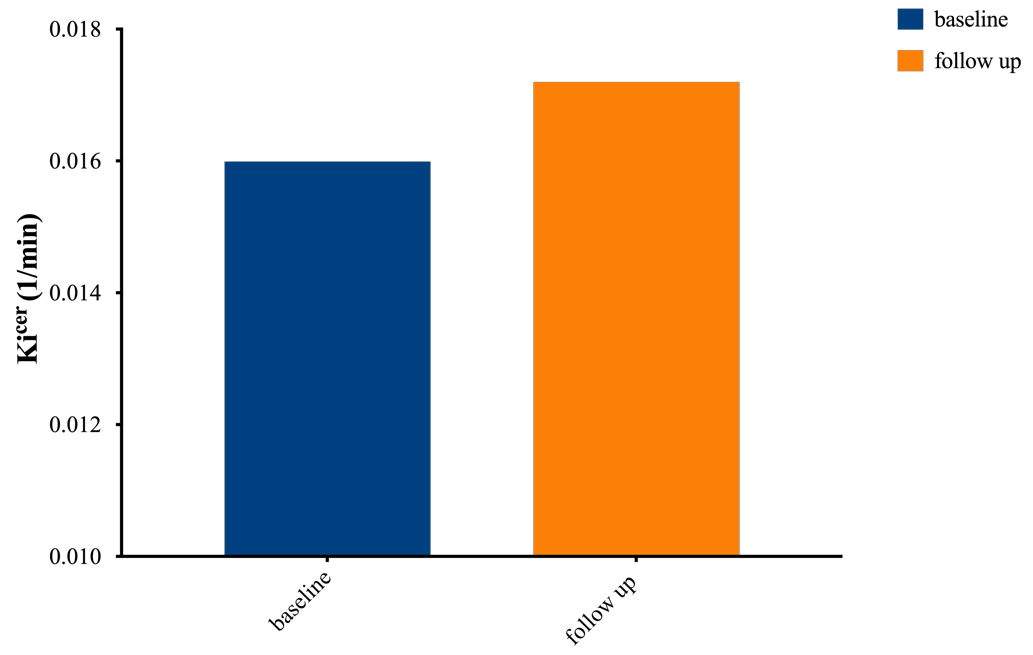


**Supplementary Tables**

**Supplementary Table 1.** Mean (SD) Ki^cer^ estimates for the whole striatum and for the striatal subdivisions by group and showing significant differences (corrected for multiple comparisons using Tukey HSD post-hoc tests).

|  | Ki^cer^ **estimates** |  |  |  | p-value |  |
| --- | --- | --- | --- | --- | --- | --- |
|  | **22q11.2 deletion group** | **Healthy controls** | **22q11.2 duplication**  **group** | **22q11.2 deletion**  **vs**  **Healthy controls** | **22q11.2**  **deletion**  **vs**  **22q11.2 duplication** | **22q11.2 duplication**  **vs**  **Healthy controls** |
| whole striatum | 0.0143  (0.0012) | 0.0127  (0.001) | 0.0119 (0.0012) | <0.001*** | <0.001*** | p=0.08 |
| Functional subdivisions |  |  |  |  |  |  |
| associative | 0.0145  (0.0013) | 0.0128  (0.0011) | 0.0119 (0.0013) | <0.001*** | <0.001*** | p=0.09 |
| sensorimotor | 0.0139  (0.001) | 0.0125  (0.001) | 0.012  (0.0013) | <0.001*** | <0.001*** | p=0.58 |
| limbic | 0.0146  (0.0013) | 0.013  (0.0011) | 0.012  (0.0014) | <0.001*** | <0.001*** | p=0.16 |

**Supplementary Table 2:** Striatal volume as quantified using regional PET volume analysis in the 22q11.2 deletion, 22q11.2 duplication and healthy control groups. One-way ANOVA showed that striatal volume was different between groups(*F*(2,55)=4.7, *p=0.013).* Using Tukey post hoc tests, we found significantly decreased striatal volume in the 22q11.2 deletion group compared to the healthy control group (p=0.021), but not between the 22q11.2 deletion and 22q11.2 duplication (p=0.99) or between the 22q11.2 duplication and healthy control groups(p=0.074), although it should be noted that in absolute terms the striatal volume in the duplication group was very similar to that in the deletion group.

|  | **striatal volume (cm^3^)**  **mean(sd)** |  |  | p-value |  |
| --- | --- | --- | --- | --- | --- |
| 22q11.2 deletion group | **Healthy controls** | **22q11.2 duplication**  **group** | **22q11.2 deletion**  **vs**  **Healthy controls** | **22q11.2**  **deletion**  **vs**  **22q11.2 duplication** | **22q11.2 duplication**  **vs**  **Healthy controls** |
| 15.74(1.6) | 17.27(2.18) | 15.74(1.6) | 0.021 | 0.99 | p=0.074 |

**Supplementary Table 3.** Sensitivity analysis: Mean (SD) Ki^cer^ estimates for the whole striatum and for the striatal subdivisions by study group after the exclusion of the individual with 22q11.2 duplication and co-morbid cerebral palsy.

|  |  | Ki^cer^ estimates |  |  |
| --- | --- | --- | --- | --- |
|  | **Individuals with 22q11.2 deletion** | **Healthy controls** | **Individuals with 22q11.2 duplication** | **p-value** |
| Whole striatum | 0.0143  (0.0012) | 0.0127  (0.001) | 0.0119  (0.0012) | <0.001*** |
| Functional subdivisions |  |  |  |  |
| associative | 0.0144(0.0013) | 0.0127(0.0011) | 0.0119 (0.0013) | <0.001*** |
| sensorimotor | 0.0139(0.001) | 0.0125(0.001) | 0.012 (0.0013) | <0.001*** |
| limbic | 0.0146(0.0013) | 0.013 (0.0011) | 0.012 (0.0014) | <0.001*** |

**Supplementary Table 4.** Mean (SD) Ki^cer^ estimates for the whole striatum and for the striatal subdivisions for the group of 22q1.2 deletion with no/minimal CAARMS positive symptoms, healthy controls and the individuals with 22q11.2 duplication.

|  |  | Ki^cer^ estimates | |  |  |
| --- | --- | --- | --- | --- | --- |
|  | **Individuals with**  **22q11.2 deletion and**  **no CAARMS symptoms** | | **Healthy controls** | **Individuals with 22q11.2 duplication** | **p-value** |
| Whole striatum | 0.0143 (0.001) | | 0.0127 (0.001) | 0.0119 (0.0012) | <0.001*** |
| Functional subdivisions |  | |  |  |  |
| associative | 0.0144(0.001) | | 0.0127(0.0011) | 0.0119 (0.0013) | <0.001*** |
| sensorimotor | 0.0139(0.001) | | 0.0125(0.001) | 0.012 (0.0013) | <0.001*** |
| limbic | 0.0146(0.0012) | | 0.013 (0.001) | 0.012 (0.0014) | <0.001*** |

**References:**

1. Kuhn FP, Warnock GI, Burger C, Ledermann K, Martin-Soelch C, Buck A. Comparison of PET template-based and MRI-based image processing in the quantitative analysis of C11-raclopride PET. *EJNMMI research* 2014; **4**(1)**:** 7.

2. Jauhar S, Veronese M, Nour MM, Rogdaki M, Hathway P, Natesan S *et al.* The effects of antipsychotic treatment on presynaptic dopamine synthesis capacity in first-episode psychosis: a positron emission tomography study. *Biological psychiatry* 2019; **85**(1)**:** 79-87.

3. Kim E, Howes OD, Veronese M, Beck K, Seo S, Park JW *et al.* Presynaptic Dopamine Capacity in Patients with Treatment-Resistant Schizophrenia Taking Clozapine: An [(18)F]DOPA PET Study. *Neuropsychopharmacology : official publication of the American College of Neuropsychopharmacology* 2017; **42**(4)**:** 941-950.

4. Froudist-Walsh S, Bloomfield MA, Veronese M, Kroll J, Karolis VR, Jauhar S *et al.* The effect of perinatal brain injury on dopaminergic function and hippocampal volume in adult life. *eLife* 2017; **6**.

5. Martinez D, Slifstein M, Broft A, Mawlawi O, Hwang DR, Huang Y *et al.* Imaging human mesolimbic dopamine transmission with positron emission tomography. Part II: amphetamine-induced dopamine release in the functional subdivisions of the striatum. *Journal of cerebral blood flow and metabolism : official journal of the International Society of Cerebral Blood Flow and Metabolism* 2003; **23**(3)**:** 285-300.
